# Estimation of sickness absenteeism among Italian healthcare workers during seasonal influenza epidemics

**DOI:** 10.1371/journal.pone.0182510

**Published:** 2017-08-09

**Authors:** Maria Michela Gianino, Gianfranco Politano, Antonio Scarmozzino, Lorena Charrier, Marco Testa, Sebastian Giacomelli, Alfredo Benso, Carla Maria Zotti

**Affiliations:** 1 Department of Public Health Sciences and Pediatrics, Università di Torino, Torino, Italy; 2 Department of Control and Computer Engineering, Politecnico di Torino, Torino, Italy; 3 AOU Città della salute e della Scienza, Torino, Italy; University of Hong Kong, HONG KONG

## Abstract

**Objectives:**

To analyze absenteeism among healthcare workers (HCWs) at a large Italian hospital and to estimate the increase in absenteeism that occurred during seasonal flu periods.

**Design:**

Retrospective observational study.

**Methods:**

The absenteeism data were divided into three “epidemic periods,” starting at week 42 of one year and terminating at week 17 of the following year (2010–2011, 2011–2012, 2012–2013), and three “non-epidemic periods,” defined as week 18 to week 41 and used as baseline data. The excess of the absenteeism occurring among HCWs during periods of epidemic influenza in comparison with baseline was estimated. All data, obtained from Hospital’s databases, were collected for each of the following six job categories: medical doctors, technical executives (i.e., pharmacists), nurses and allied health professionals (i.e., radiographers), other executives (i.e., engineers), nonmedical support staff, and administrative staff. The HCWs were classified by: in and no-contact; vaccinated and unvaccinated.

**Results:**

5,544, 5,369, and 5,291 workers in three years were studied. The average duration of absenteeism during the epidemic periods increased among all employees by +2.07 days/person (from 2.99 to 5.06), and the relative increase ranged from 64–94% among the different job categories. Workers not in contact with patients experienced a slightly greater increase in absenteeism (+2.28 days/person, from 2.73 to 5.01) than did employees in contact with patients (+2.04, from 3.04 to 5.08). The vaccination rate among HCWs was below 3%, however the higher excess of absenteeism rate among unvaccinated in comparison with vaccinated workers was observed during the epidemic periods (2.09 vs 1.45 days/person).

**Conclusion:**

The influenza-related absenteeism during epidemic periods was quantified as totaling more than 11,000 days/year at the Italian hospital studied. This result confirms the economic impact of sick leave on healthcare systems and stresses on the necessity of encouraging HCWs to be immunized against influenza.

## Introduction

The annual occurrence of seasonal flu epidemics and subsequent work absenteeism, coupled with the low immunization coverage achieved among healthcare workers (HCWs), may have a significant impact on patient health, requiring targeted policy interventions.

The WHO has estimated that as a result of seasonal influenza epidemics, 5–15% of the population is affected by upper respiratory infections, and 3–5 million cases of severe illness and between 250,000 and 500,000 deaths occur each year worldwide [1].

Globally, access to vaccination is considered insufficient in many populations, including high-risk groups. Moreover, the WHO objective of achieving vaccination coverage of at least 50% by 2006 and 75% by 2010 in the elderly population and among at-risk individuals was not met [2].

A large study conducted in Europe [3] reported vaccination rates in the general population ranging from 10–30%, with the lowest rate of vaccination identified in those under 50 years of age. Additionally, in elderly subjects (≥65 years), vaccination rates ranged from a minimum of 14% (Ireland) to a maximum of 70% (UK). Thus, immunization rates were low, even among patients with chronic respiratory or cardiovascular diseases (25–60%) and the elderly (17–90%).

Low immunization among HCWs is a major issue both because of the risk of transmitting vaccine-preventable infections to patients, and especially those at high risk, and given the need to maintain high health personnel availability during epidemics. In healthcare settings, influenza, which is spread by droplet transmission, may be introduced by visitors, patients, and staff, with serious consequences for elderly and immunocompromised patients, and patient isolation may be insufficient to contain influenza transmission [4]. Available data demonstrate that despite 30 years of official recommendations, the immunization rate among HCWs in Europe rarely exceeds 30–40% [3].

In Italy, between 2000 and 2015, vaccination coverage in the general population ranged from 13–19%. The coverage of the elderly population (the first target of vaccination) exceeded 65% only a few times between 1999 and 2015, while among HCWs, the data regarding coverage during the pandemic period (2009–2010) suggested vaccination coverage of only 15%.

Although there has been some general interest in analyzing the correlation between work absenteeism (in the general population as well as among HCWs) and influenza epidemics, obtaining the data necessary to allow for precise quantification is difficult; this difficulty mainly derives from challenges in obtaining comparable data due to different policies for recording work absenteeism in different countries, leading to different levels of sensitivity and specificity.

After the 2009 influenza pandemic, several publications compared work absenteeism related to the pandemic with absenteeism during periods of seasonal epidemics. One study of absenteeism among HCWs in Hong Kong [5] during a seasonal epidemic highlighted 8.4% and 26.5% excesses in absenteeism due to any cause and respiratory diseases, respectively. Moreover, during the study period (2004–2009), the average durations of an absence from work due to illness overall and respiratory illness in particular amounted to 2.3 days and 1.39 days, respectively. Meanwhile, a Canadian study evaluating the general population estimated a 12% increase in absenteeism per year due to the seasonal flu, with an average loss of 14 working hours per worker [6]. Another study conducted on workers aged over 50 years in the USA reported an average loss of 1.3 working days due to influenza-like illness (ILI) [7]. Finally, a study conducted in the UK [8] estimated a 10–12% increase in HCW absenteeism due to influenza or ILI.

A systematic review published in 2014 highlighted the effectiveness of HCW vaccination against influenza in significantly reducing mortality from all causes and ILI [9]. Nevertheless, only a few studies of good quality and at low risk of bias have determined vaccination’s preventive efficacy and its effective impact on absenteeism [10–17]. Additionally, the economic impact that a preventive vaccination campaign could have on reducing work absenteeism is a topic of interest for both researchers and national healthcare systems.

The aims of the present study were to analyze absenteeism among HCWs at a large Italian hospital and to estimate the increase in absenteeism that occurred during seasonal flu periods.

## Materials and methods

### Design

The AOU “*Città della salute e della Scienza*” in Turin is a complex of four interconnected hospitals (Molinette, OIRM, S. Anna, and CTO) with more than 1,700 beds and is the main teaching hospital of the University of Turin’s School of Medicine. We conducted this study based on data from Molinette, which has approximately 5,500 workers, accounting for approximately 45% of the center’s employees.

In this study, we analyzed data from the three consecutive years following the influenza pandemic of 2009, during which seasonal influenza outbreaks were of medium intensity.

Absenteeism data were obtained from the hospital’s Personal Unit Database and included the number of days of paid sick leave during the periods of July 2010 to June 2011, July 2011 to June 2012, and July 2012 to June 2013. Such database also comprised for every employee a set of attributes like as personal data, job category, position, work place.

From the Occupational Health Unit of Molinette Hospital we obtained data on influenza vaccination for each employee and we merged this database with the Personal Unit Database in order to obtain a single, comprehensive database.

We focused on “sporadic absences,” defined as unplanned sickness absenteeism due to any cause.

We could not obtain a dataset including only ILI-related and acute respiratory infection (ARI)-related absences because based on the Italian policy regarding absenteeism records in the workplace, it is not compulsory to note the medical diagnosis reported on the sickness absence certificate issued by the medical practitioner.

We divided the data from the different years into three “epidemic periods,” starting at week 42 of one year and terminating at week 17 of the following year (2010–2011, 2011–2012, and 2012–2013), and three “non-epidemic periods,” which were defined as week 18 to week 41 and used as baseline data. The epidemic period counted 196 days and the non-epidemic period counted 168 days. During these three consecutive years that followed the influenza pandemic of 2009, outbreaks of moderate intensity occurred with the following ILI epidemic incidence rates:

- 2010–2011: 103/1,000 person-years;
- 2011–2012: 86/1,000 person-years;
- 2012–2013: 105/1,000 person-years.

Data for Italian influenza epidemics were obtained from Influnet, the Italian sentinel influenza surveillance network. Influnet specifically comprises organized networks of primary care physicians, and mostly general practitioners (GPs), covering at least 1–5% of the population. Between week 42 and week 17, sentinel physicians reported the weekly number of patients with ILI, ARI, or both to the national center for influenza surveillance [18].

In this study, we also compared ILI morbidity data for the three evaluated seasonal epidemic periods (provided by the regional epidemiological service (SEReMI)) with absenteeism rates at the target hospital during the same periods.

Individual sickness absenteeism data were grouped for each of the following job categories:

1. medical doctors;
2. technical executives (i.e., pharmacists, dieticians, and chemists);
3. nurses and allied health professionals (i.e., radiographers, therapists, and laboratory technicians);
4. other executives (i.e., engineers, lawyers, analysts, and statistical and administrative staff);
5. nonmedical support staff (i.e., ward assistants and cleaning staff);
6. administrative staff.

The overall personnel were also grouped into two categories (in-contact and no-contact), depending on the nature of their work relationship with patients. The “in-contact” category included all workers who were engaged in direct contact with patients during admission, diagnosis, treatment, and/or follow-up. The “no-contact” category included all workers who did not work in proximity to patients and who therefore had a lower risk of transmitting disease.

The study protocol was approved by the Directorate-General of AOU (November 26, 2013).

### Statistical analysis

We developed a general and robust R [19] pipeline that was able to process the input data through the following steps:

- input preparation;
- descriptive analysis;
- comparison with reference data;
- stratification and risk analysis;
- output.

#### Input preparation

One set of csv files was input into the pipeline per solar year. In these files, each row represented an employee, and each column described one of several attributes used for stratification (e.g., position, contact with patients, sex) and the number of days of absence during each of the 52 weeks of the year. The data were also pre-processed by merging two files at a time and extracting representative data for a given flu-year (i.e., the flu-year starts from week 27 of a given year and lasts until week 26 of the next year). This transformation allowed us to better assess the dynamics of a single flu epidemic whose peak occurred during the winter and spanned across years.

#### Descriptive analysis

The pre-processed data were then descriptively analyzed. For each stratification variable, we computed its frequency distribution within the available classes. Afterward, the pipeline produced graphical output to visually describe the trends in the flu absenteeism occurring over the years analyzed. To better interpret the data, the trends were interpolated by fitting a lowess curve that smoothed the intra-week variance due to seasonal vacations and other possible sources of absenteeism (i.e., parental care and other reasons for sickness).

#### Comparison with reference data

To increase data comparability between different populations that were most likely heterogeneous in terms of distribution, the results were represented in terms of days lost per person due to illness.

If reference data were available (i.e., regional/government observational flu data), the pipeline was able to graphically produce a comparison between the experimental and reference data. In our case, the reference data showed a cleaner signal, mainly because of the high specificity of these data (only representing certified ILI or ARI cases). In contrast, the experimental data exhibited a higher level of noise, which resulted in a greater basal offset and, consequently, a more compressed signal.

#### Stratification and risk analysis

After the descriptive analysis, the pipeline employed the *epiR* [20] and *meta* [21] packages to compute a risk analysis score for each flu year and a cumulative meta-analysis score to determine the average intra-year risk of absenteeism due to the flu.

Risk analysis (RA) is a widely addressed method in epidemiology to identify and quantify, in terms of numerical probability, the relationship (risk) between the exposure to a given condition (flu period/non-flu period) and a given effect (absenteeism /no absenteeism).

In the proposed study, individuals in the flu period (the "case" group) are compared with individuals in the non-flu period (the "control" group). We constructed a 2×2 confusion matrix (Table 1) representing the count of absenteeism days of cases (A), absenteeism days of controls (B), no absenteeism days of cases (C) and no absenteeism days of controls (D).

**Table 1:** 2x2 confusion matrix with cumulative incidence (CI).

	ABSENTEEISM DAYS	NO ABSENTEEISM DAYS	CI
flu period (196 days)^a^	A	C	CI_exposed_
non-flu period (168 days)^a^	B	D	CI_unexposed_

^*a*^ The duration of the flu and non-flu periods is counted on the basis of epidemiologic and virological surveillance by general practitioners

From the confusion matrix we computed the cumulative incidence (CI), which is an estimate of the risk of absenteeism for each exposure group and is computed as follow:

$${\text{CI}}_{\text{exposed}}=\text{ A}/\text{A}+{\text{C and CI}}_{\text{unexposed}}=\text{ B}/\text{B}+\text{D}.$$

Furthermore, in order to retain a more robust overall insight into absenteeism risk over the three-year timespan, we applied a meta-analysis approach by correcting with fixed-effect estimate, using the Mantel-Haenszel method [22], to properly manage differences in population across the three years timespan.

#### Output

In order to elucidate the difference in days of a condition between an exposed and an unexposed population we resorted to the following mathematical expression:

$$\text{Excess absenteeism }=\;(\text{epidemic days }*{\text{CI}}_{\text{exposed}})\;-(\text{non epidemic days }*{\text{CI}}_{\text{unexposed}}).$$

The Excess absenteeism score conservatively quantified the flu effect in terms of the excess of absenteeism days observed during the flu periods and for years/person.

## Results

The numbers of HCWs employed at the target hospital were 5,544, 5,369, and 5,291 during the three years under study (2010, 2011, and 2012, respectively; 73% female). The reduction observed in the number of workers was predominantly caused by retirement, no data were missing.

In the three years under analysis, there were no significant differences in the distribution of workers by job category, age class, percentage of employees working in direct contact with patients, or vaccination rate. Most employees were nurses and allied health professionals, were aged between 40 and 59 years, and worked in direct contact with patients, and only 2.2–2.9% of employees were vaccinated (Table 2).

**Table 2:** Population characteristics.

	2010–2011	2011–2012	2012–2013
**Female**	4,024 (72.6%)	3,925 (73.1%)	3,872 (73.2%)
**Age (years)**	40–49 (37.2%)	40–49 (37.7%)	40–49 (38.2%)
Job category*			
Medical doctors	814 (14.7%)	787 (14.7%)	771 (14.6%)
Technical executives	103 (1.9%)	100 (1.9%)	97 (1.8%)
Nurses and allied health professionals	2,537 (45.8%)	2,465 (45.9%)	2,454 (46.4%)
Other executives	30 (0.5%)	30 (0.6%)	30 (0.6%)
Nonmedical support staff	1,306 (23.6%)	1,258 (23.4%)	1,230 (23.2%)
Administrative staff	754 (13.6%)	729 (13.6%)	709 (13.4%)
**Job in contact with patients**			
Yes	4,753 (85.7%)	4,603 (85.7%)	4,535 (85.7%)
**Vaccination**			
Yes	159 (2.9%)	158 (2.9%)	117 (2.2%)

* Medical doctors: i.e., physicians and radiologists, Technical executives: i.e., pharmacists, dieticians, biologists, chemists, and similar professions, Nurses and allied health professionals, i.e., radiographers, therapists, and laboratory technicians, Other executives: i.e., engineers, lawyers, analysts, and statistical and administrative staff, Nonmedical support staff: i.e., ward assistants and cleaning staff

**Fig 1:**
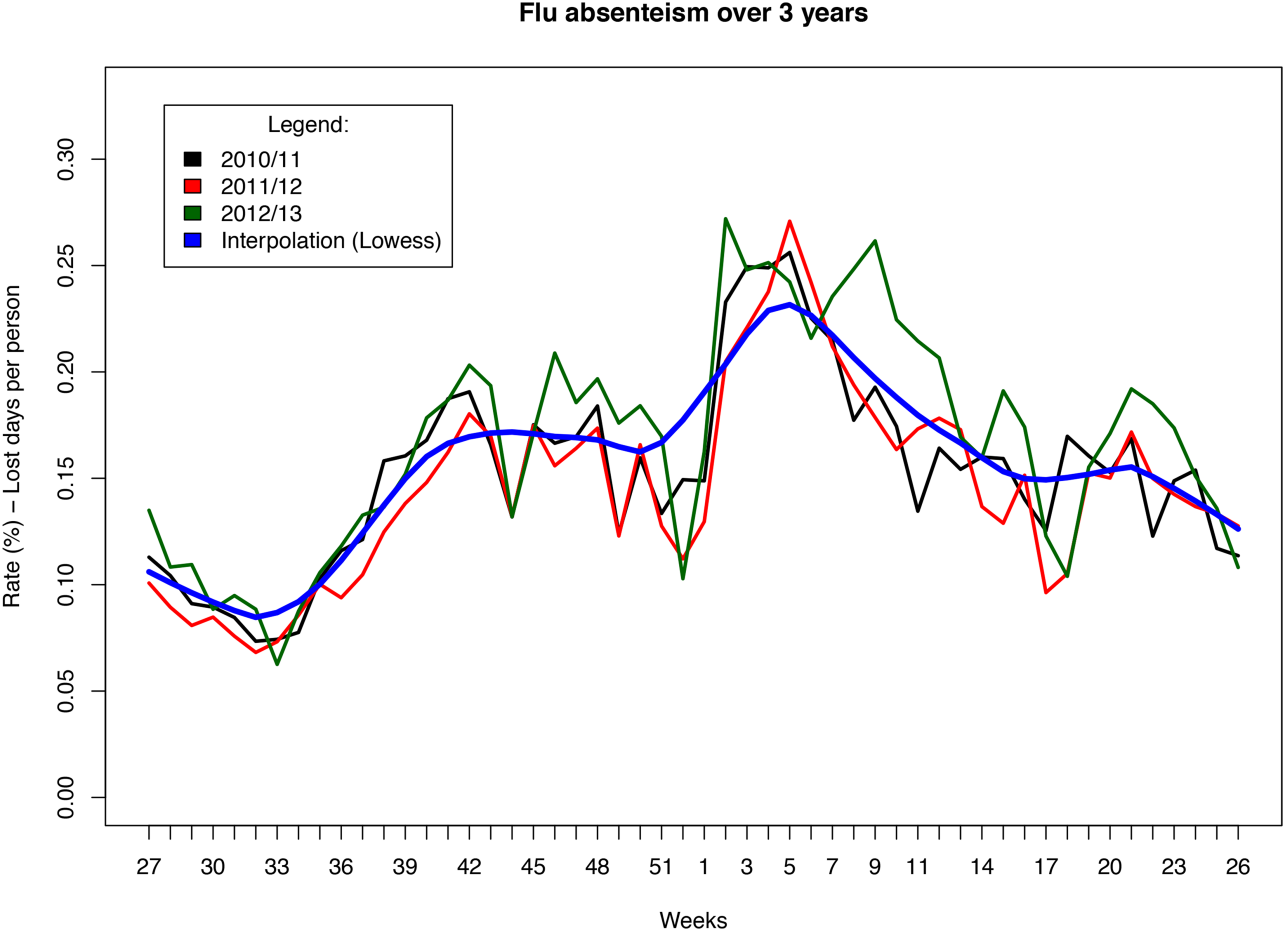
Weekly sickness absenteeism rates among HCWs.

**Fig 2:**
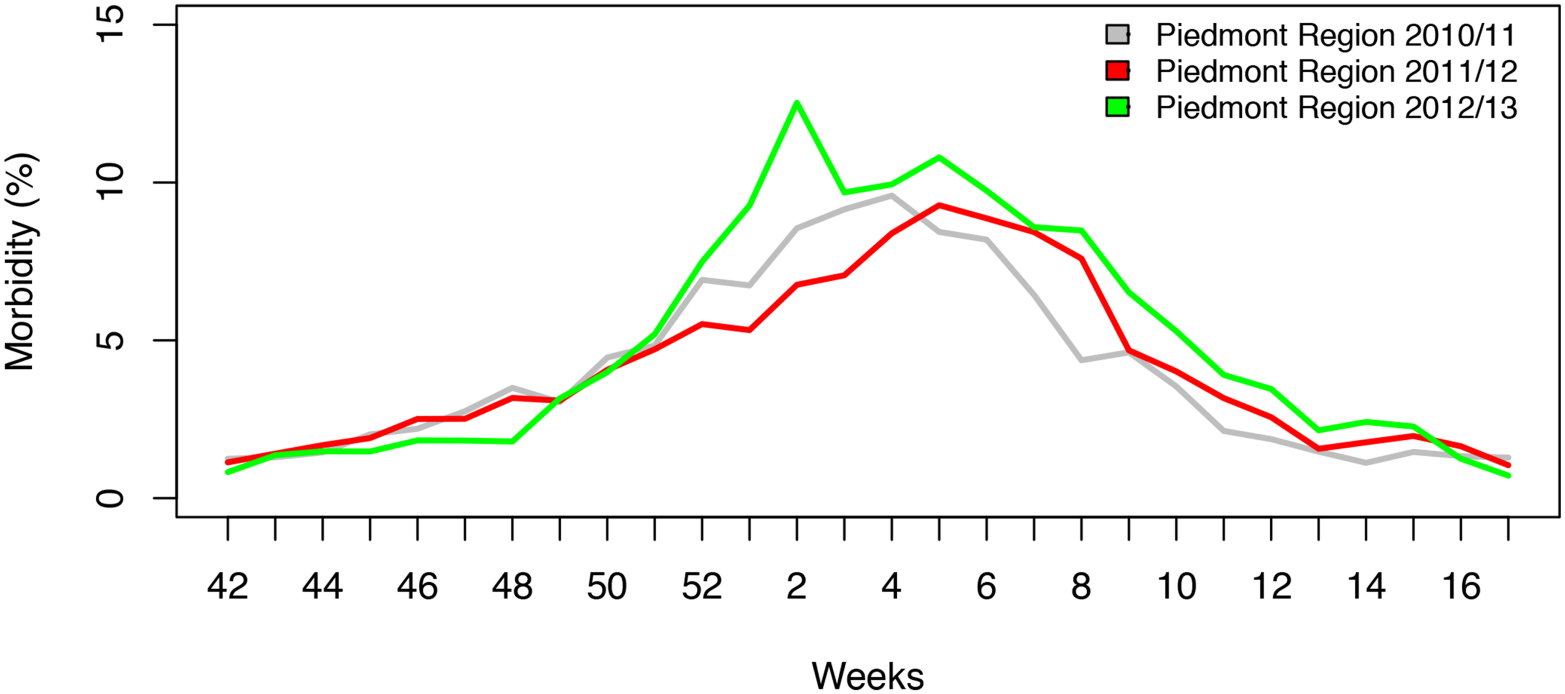
Morbidity rates associated with influenza epidemics in the Piemonte region.

Fig 1 shows that the sickness absenteeism rates in the Piemonte region increased during each of the epidemic periods, and there were discernible peaks between the 51^st^ week and the 11^th^ week in 2010–2011 and between the 52^nd^ week and the 14^th^ week in both 2011–2012 and 2012–2013. Further supporting the hypothesis that the peaks in absenteeism were related to the influenza epidemic, Fig 2 shows that during the same period, a similar trend was observed in ILI morbidity in the local community within the Piemonte region.

Given that the vaccination rate among HCWs was below 3%, the overall trend in absenteeism was not significantly affected by the inclusion or exclusion of vaccinated workers.

The average duration of absenteeism during the epidemic period increased among all employees by +2.07 days/person (from 2.99 to 5.06 days/person).

Table 3 shows the increase in sick leave during the epidemics among employees in each job category, with the relative increase ranging from 64–94% among the different types of staff.

**Table 3:** Mean rates of absenteeism during different periods and excess absenteeism during epidemic periods (working days lost per person per year).

Characteristic	Non-epidemic periods	Epidemic periods	Excess absenteeism (years/person)	p-value
**Job category**				
Medical doctors	0.58	1.04	0.45	p<0.01
Technical executives	0.98	1.91	0.92	p<0.05
Nurses and allied health professionals	2.75	4.70	1.95	p<0.01
Others executives	0.56	0.91	0.36	p<0.01
Nonmedical support staff	5.17	8.57	3.40	p<0.01
Administrative staff	3.07	5.22	2.15	p<0.01
**Job in contact with patients**				
Yes	3.04	5.08	2.04	p<0.01
No	2.73	5.01	2.28	p<0.01
**Vaccination**				
Yes	0.45	1.90	1.45	p<0.01
No	3.07	5.16	2.09	p<0.01

In comparison with other job categories, the absolute increases in absenteeism were highest among nonmedical support staff (+3.4), administrative staff (+2.15) and nurses and allied health professionals (+1.95). These categories also had higher levels of absenteeism during non-epidemic periods (5.17, 3.07, 2.75 days, respectively) in comparison with other categories.

The ranking of the absenteeism rates by job category during non-epidemic and epidemic periods were comparable.

Workers not in contact with patients experienced a slightly greater increase in absenteeism than did employees in contact with patients. The absolute value of the observed excess absenteeism was 2.28 days/person, which was equivalent to an excess of approximately 84% in relative terms. Workers not in contact with patients exhibited lower rates of absenteeism during non-epidemic periods in comparison with workers in contact with patients (2.73 vs 3.04 days/person).

The absenteeism rate among workers without vaccination during the epidemic periods was approximately 1.5 times higher than the rate observed among vaccinated employees. The absolute and relative increases were 2.01 days/person and approximately 70%, respectively.

## Discussion

Our study showed that there was an increase in absenteeism among hospital workers during periods of epidemic influenza in Italy (average of +2.07 days/person). Compared with the average of absenteeism during non-epidemic periods, used as baseline data, this absolute increase correlated with a relative increase of 70% (from 2.99 to 5.06 days/person).

This finding is in agreement with the results of previous studies. In 1980–1981, an epidemic of influenza A in Winnipeg resulted in a nearly 2-fold increase in work time lost by hospital workers (nurses and support personnel) compared with data for the remainder of the year excluding the epidemic period [23]. In Canada, another study demonstrated that there was a significant difference between the absenteeism rates among employees in high-risk departments during the 1987–1988 influenza season and the non-influenza season during the same year, with an approximately 35% higher absenteeism rate observed during the influenza season [24].

In contrast, a study in Hong Kong based on data collected over a six-year period found a modest increase in absenteeism among HCWs during influenza epidemics (+8.4%). Finally, during two epidemics (1993–1994 and 1996–1997), another study conducted in the UK detected only a limited change in the rates of sickness absenteeism [25].

These discrepant findings could be explained by differences in the intensity, frequency, and duration of the epidemics; the strains of influenza virus involved in the epidemics; or the methodology employed in the studies, such as the study period evaluated, the staff type analyzed and the size of the sample population of workers, all of which may impact the sickness absenteeism estimates. Nevertheless, our results, which were supported by morbidity trends in the Piemonte region, very likely represent the typical impact of seasonal influenza in Italy.

The rate of absenteeism observed in this study increased significantly within all job categories during epidemic periods. An important factor to consider when quantifying the impact of influenza epidemics on HCWs is the relationship between employment role (i.e., medical doctors, technical and other executives, nurses and allied health professionals, nonmedical support staff, and administrative staff) and the length of the sick leave. In our study, employees belonging to the first three job categories returned to work, on average, after less than two days of sick leave during epidemic periods, whereas the workers in the other categories had an average sick leave duration of approximately five days. The comparison between the epidemic and non-epidemic periods showed that the increase in absenteeism during epidemic periods was lower among workers in the first three categories (on average less than 1 day/person) than among workers in the other three categories (on average more than 2 days/person); this finding may be attributable to the fact that the medical and executive personnel (approximately 17% of personnel) may recognize that the hospital cannot obtain coverage for their positions when they take sick leave and therefore may feel obligated to return to work as early as possible.

We hypothesized that due to the low vaccination coverage that characterizes Italian HCWs (such as those included in the study), vaccination has not had a substantial impact on work absenteeism trends, and our results confirm this hypothesis. Nevertheless, our study showed that unvaccinated employees used approximately 3.2 days of additional sick leave per person during the influenza season compared with vaccinated employees. Interestingly, the difference in absenteeism between vaccinated and unvaccinated HCWs was also evident also during the non-epidemic periods, with unvaccinated HCWs having, on average, 2.5 additional days/person lost due to sick leave. This discrepancy may be explained by the possibility that vaccines also protect employees from illness during non-epidemic periods. Moreover, the greater difference in absenteeism observed during the epidemic periods supports the beneficial effect of influenza vaccination on reducing absenteeism, as observed in previous studies [17, 26].

These results could be useful to populate mathematical models that allow to estimate the effects of vaccination interventions; the experiences of models developed [27,28] are useful for describing the changes in the dynamics of influenza in relation to the behavioral changes of the affected population (vaccinations, preventive measures). Stationary pattern or time-space transitions can also be described in predictive mathematical models and absenteeism data can help to read both behavioral and economic changes.

In the present study, the rates of absenteeism were slightly higher during both epidemic and non-epidemic periods among workers in direct contact with patients. However, during the epidemic periods, there was a slightly lesser increase in the use of sick leave among workers having direct contact with patients relative to those with no patient contact. Although influenza is a community infection, so contact with patients may not be its main mode of transmission, the results showed that HCWs with direct patient contact were at increased risk of becoming infected and experiencing absenteeism year-round.

This study has some limitations. First, the use of the data referring to sporadic absences, defined as unplanned sick leaves due to any cause, and no data referring only ILI-related and acute respiratory infection (ARI)-related absences, might be a limitation. The performance of similar analyses using GP-certified sick leave data may lead to more robust estimates. However the results reported in this work are statistically valid and the hypothesis that the peaks in absenteeism were related to the influenza epidemic, is supported by the fact that during the same period, a similar trend was observed in ILI morbidity in the local community within the Piemonte region.

Second, our study analyzed data from a database, and not a prospective cohort however we utilized data collected over 3 years, and our study sample covered a very large population of HCWs spanning all job categories.

In conclusion, at Molinette Hospital, considering the average of +2.07 days/person and all employees, influenza-related absenteeism during the considered epidemic periods was quantified as totaling more than 11,000 days/year”. This result confirms the economic impact of influenza-related absenteeism on healthcare systems. The difference in sickness absenteeism between vaccinated and unvaccinated HCWs stresses the necessity of encouraging HCWs to be immunized against influenza.
